# Acid protonation promoted different crystal phase structure silicon carbide-based carbon nitride composites to enhance the photocatalytic degradation of dye wastewater

**DOI:** 10.1039/d3ra06438g

**Published:** 2023-12-07

**Authors:** Zhiqiang Wu, Yueyi Li, Xuesong Li, Enke Feng, Lin-an Cao, Zhenliang Li, Xuming Wang, Pengxi Jiang, Dailian Wang

**Affiliations:** a School of Chemistry and Chemical Engineering, Key Laboratory of Green Catalytic Materials and Technology in Ningxia, Ningxia Normal University Guyuan 756000 P. R. China wuzqns@nxnu.edu.cn wdl1731624323@163.com; b School of Civil and Hydraulic Engineering, Ningxia University Yinchuan 750021 P. R. China; c School of Physics and Electronic and Electrical Engineering, Ningxia University Yinchuan 750021 P. R. China

## Abstract

Acid-protonated crystalline silicon carbide-supported carbon nitride photocatalytic composites were successfully prepared by the impregnation-heat treatment method (P-g-C_3_N_4_/β-SiC and P-g-C_3_N_4_/α-SiC). The samples were characterized by X-ray diffraction (XRD), scanning electron microscopy (SEM), transmission electron microscopy (TEM), X-ray photoelectron spectroscopy (XPS), Fourier transform infrared (FT-IR), UV-vis diffuse reflectance spectra (UV-vis-DRS) photoluminescence (PL), *etc*. The results of SEM showed that the P-g-C_3_N_4_/β-SiC and P-g-C_3_N_4_/α-SiC materials were transformed from large-area lamellar structures to uniform and dispersed lamellar particles. The UV-vis-DRS and PL showed that the recombination probability of photogenerated electron–hole pairs of P-g-C_3_N_4_/β-SiC and P-g-C_3_N_4_/α-SiC samples decreased and the band gap increased. The results of photocatalytic degradation of alizarin red S (ARS), acid fuchsin (AF), and basic fuchsin (BF) showed that the samples P-g-C_3_N_4_/β-SiC and P-g-C_3_N_4_/α-SiC had excellent photocatalytic degradation performance. It is worth noting that the degradation performance of the sample P-g-C_3_N_4_/β-SiC on the three dyes is better than that of P-g-C_3_N_4_/α-SiC. The electron spin resonance spectra (ESR) results showed that the ˙O_2_^−^ and ˙OH produced by the two catalysts during the dye degradation process played a leading role in the degradation reaction. Fortunately, the catalyst maintains an excellent cycle life and can be reused more than seven times while degrading all three dyes.

## Abstract 2

Acid-protonated crystalline silicon carbide-supported carbon nitride photocatalytic composites were successfully prepared by the impregnation-heat treatment method (P-g-C_3_N_4_/β-SiC and P-g-C_3_N_4_/α-SiC).

## Graphical abstract

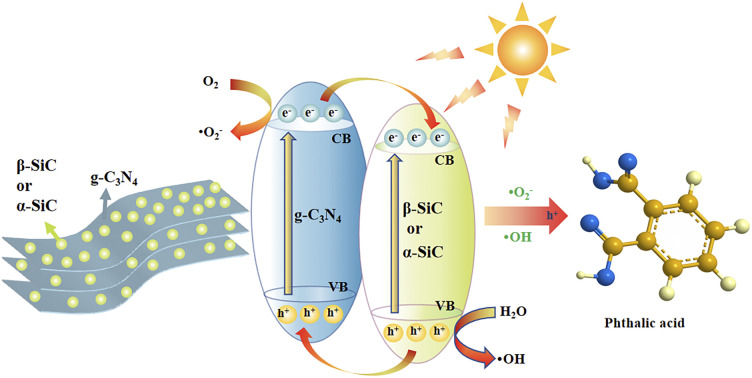

## 1. Introduction

Currently, with the globalization of the economy and the rapid development of industrial society, the problems of energy crisis and environmental pollution have become increasingly prominent. Semiconductor photocatalytic methods have attracted wide attention because of their sustainable development and environmental friendliness characteristics.^1,2^ It is the current goal to seek a low-cost and environmentally friendly semiconductor photocatalyst suitable for mass production, that is sensitive to visible light.^3,4^ Common semiconductor photocatalytic materials include: metal oxide photocatalytic materials (such as TiO_2_,^5^ ZnO,^6^*etc.*); Metal sulfides (CdS,^7^*etc.*); Bi-based photocatalysts (BiVO_4_,^8^*etc.*); and non-metallic semiconductor catalysts (g-C_3_N_4_,^9^*etc.*). However, some metal semiconductor photocatalysts, such as TiO_2_ and ZnO, have a large band gap,^10^ which hinders the utilization of visible light. The bismuth-based photocatalyst has good visible light utilization, stable chemical properties and moderate conduction and valence band positions, which obviously solves the visible light absorption problem of TiO_2_. However, low quantum efficiency and photogenerated carrier recombination is still an urgent problem for bismuth-based photocatalysts.^11^ CdS, *etc* are representative materials of sulfides in the field of photocatalysis, with adjustable energy bands, but when they change from multilayer to single-layer, their band gap widens, and their optical and electrical properties also change. The photocatalytic performance of these semiconductor materials is not high.^12^ Therefore, in order to solve the above existing problems, metal-free semiconductors have attracted great attention from scientists. In the past decade, graphite phase carbon nitride (g-C_3_N_4_), a typical inorganic non-metallic polymer semiconductor, has been widely used in photocatalytic hydrogen production, organic pollutant degradation, CO_2_ emission reduction, and photocatalytic organic synthesis due to its chemical stability, rich raw materials, and simple preparation.^13–16^ However, the bulk g-C_3_N_4_ has low photocatalytic performance due to its inherent low quantum efficiency, narrow spectral response, small specific surface area and low electrical conductivity. Therefore, researchers are committed to developing various methods to improve the photocatalytic performance of materials, such as metal or non-metallic element doping,^17,18^ supported promoters,^19^ precursor pre-treatment to control morphology^20^ or construction of heterojunctions.^21^ According to previous reports, non-metallic doping will produce a recombination center of photogenerated electron holes in the catalyst, resulting in a decrease in the number of carriers and photocatalytic activity.^22^ Metal leakage caused by metal doping will cause secondary pollution to the environment. Therefore, the construction of heterojunctions has become a research focus of modified g-C_3_N_4_ materials.^23^ Heterojunction is a composite of photocatalytic materials formed by combining with other semiconductors or metals with similar bandgaps. The problem of recombination of electron hole pairs can be reduced.^24^ Generally, the g-C_3_N_4_ is coupled with different kinds of inorganic semiconductors, such as TiO_2_,^25^ CdS^26^ or SiC,^27^*etc*.

Silicon carbide is an economical, inexpensive and widely used inorganic material, mainly including two crystal types α-SiC and β-SiC, respectively. As a semiconductor, silicon carbide has unique properties,^28,29^ and its excellent catalytic properties are mainly used in photohydrolysis for hydrogen production, energy storage, *etc*. It has been reported that materials with good photocatalytic properties are prepared by compounding silicon carbide and carbon nitride, mainly used in applications such as photocatalytic hydrolysis of hydrogen production and degradation of organic pollutants.^27,30,31^ The energy bands of silicon carbide and graphitic carbon nitride can be well matched. They can form a type-II heterojunction, which is beneficial to the separation and transport of photogenerated electron–hole pairs.^32^ For type-II heterojunctions, theoretically, electrons can be transferred from g-C_3_N_4_ to SiC after the formation of electron hole pairs under light conditions, and the photogenerated holes move in the opposite direction, thus realizing the spatial separation of photogenerated electron holes.^33^ In 2017, Huang. F. *et al.* first supported^27^ silicon carbide on the surface of carbon nitride and carried out researched on the photocatalytic hydrogen production performance of g-C_3_N_4_/SiC. The results show that the maximum BET of g-C_3_N_4_/SiC material is 15.0 m^2^ g^−1^. The hydrogen production capacity of the photocatalyst is 182 μmol g^−1^ h^−1^, which is 3.4 times that of pure carbon nitride. Nanomaterialization is also one of the essential ways to improve the specific surface area of photocatalysts, control the morphology, adjust the electronic properties and modify the surface functionalization. Gan Z. X *et al.* designed^34^ a new metal-free composite material of SiC nanoparticles and g-C_3_N_4_ nanosheets by constructing a type II heterostructure. They applying them to the degradation of methyl orange (MO) wastewater. The results showed that the photocatalytic performance of g-C_3_N_4_ nanosheets was significantly improved after being modified by SiC nanoparticles. The degradation rate of MO within 1 h was 55%, which was higher than that of pure SiC and g-C_3_N_4_ catalysts under the same conditions. In 2023, Guan Y *et al.* constructed 0D/1D/2D type Z-heterojunction of Ag nanodots/SiC nanofibers/g-C_3_N_4_ nanosheets for efficient photocatalytic water decomposition;^35^ Guo H. H *et al.* prepared a Z-type heterostructure SiC/g-C_3_N_4_ composite material with enhanced photocatalytic degradation of tetracycline under visible light and the degradation rate reached 71.1% in 180 min.^36^ Similarly, studies on silicon carbide and carbon nitride composite catalysts have been reported, mainly including g-C_3_N_4_-SiC-Pt,^37^ ZrB_2_-SiC/C_3_N_4_.^38^ Its applications mainly include the degradation of wastewater such as rhodamine B, alizarin red, and tetracycline, photocatalytic hydrogen production, and the construction of pressure-resistant materials. However, the SiC used in previous research reports are all commercial micro- or nano-powders, and its crystal form is a mixed crystal phase of α-SiC and β-SiC. Moreover, in these studies, one is the lack of composite material design and performance comparison test of pure nano-sized crystalline phase SiC and g-C_3_N_4_; the other is that the specific surface area of the composite material prepared is relatively small, which greatly limits the catalytic performance of the material. Meanwhile, because of the existence of two different crystal forms of silicon carbide, based on the previous research,^39,40^ we considered composite graphite phase carbon nitride with two other crystal structures of silicon carbide, and protonated them through acid to study their differences in photocatalytic performance.

In this work, the acid protonated nanolayered composite photocatalyst P-g-C_3_N_4_/α-SiC and P-g-C_3_N_4_/β-SiC were successfully synthesized. In addition, the catalyst was applied to the photocatalytic degradation of alizarin red, acid fuchsin and basic fuchsin dye wastewater, and its degradation efficiency was excellent. The characterizations of ESR have proved that the effective catalytic activity factors of the catalyst for degrading dye wastewater are mainly superoxide radicals and hydroxyl radicals, while the role played by holes is almost negligible.

## 2. Results and discussion

**Fig. 1:**
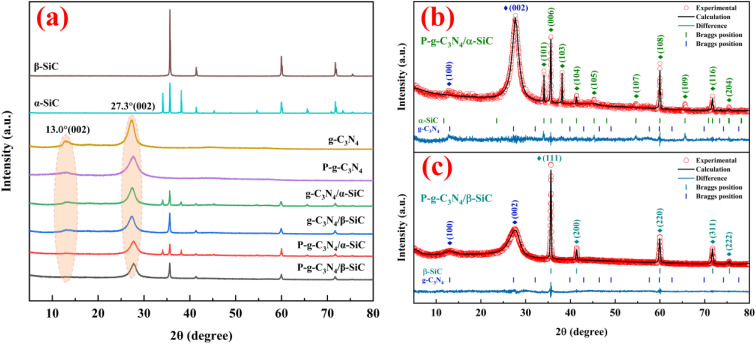
The XRD spectra of α-SiC, β-SiC, g-C_3_N_4_, P-g-C_3_N_4_, g-C_3_N_4_/α-SiC, g-C_3_N_4_/β-SiC, P-g-C_3_N_4_/α-SiC and P-g-C_3_N_4_/β-SiC, respectively (a), Rietveld refinement results of P-g-C_3_N_4_/α-SiC (b) and P-g-C_3_N_4_/β-SiC (c).

The XRD patterns of α-SiC, β-SiC, g-C_3_N_4_, g-C_3_N_4_/α-SiC, g-C_3_N_4_/β-SiC, P-g-C_3_N_4_/α-SiC and P-g-C_3_N_4_/β-SiC synthesized samples are shown in Fig. 1a. The Rietveld refinement results of P-g-C_3_N_4_/α-SiC and P-g-C_3_N_4_/β-SiC synthesized samples are shown in Fig. 1(b) and (c), respectively. And the detailed diagram shows that the peak positions of β-SiC are at 35.6°, 41.4°, 60.0°, 71.8°, and 75.5°, corresponding to the (111), (200), (220), (311) and (222) crystal planes of β-SiC,^41^ respectively. The leading peak positions of α-SiC are at 34.1°, 35.6°, 38.1°, 41.4°, 45.3°, 54.6°, 65.6°, 71.8° and 75.5° corresponding to the (101), (006), (103), (104), (105), (107), (109), (116) and (204) crystal planes of α-SiC. The diffraction peaks of g-C_3_N_4_ appearing at 13.0° and 27.4° correspond to the (100) and (002) diffraction planes.^42^ The g-C_3_N_4_/SiC (α-SiC and β-SiC) samples prepared by high-temperature sintering retained independent diffraction peaks, and no new diffraction peaks appeared, indicating that the combination of g-C_3_N_4_ and SiC would not affect the respective crystal structures. However, the diffraction peaks of g-C_3_N_4_/α-SiC and g-C_3_N_4_/β-SiC at 13.0° and 27.4° are significantly weaker than those of the bulk g-C_3_N_4_, which may be due to the uniform deposition of SiC that weakens the characteristic diffraction peaks of g-C_3_N_4_. In addition, after the samples g-C_3_N_4_/SiC were protonated by HCl, the diffraction peak intensity with a 2*θ* of 13.1° changed significantly. This peak almost disappeared, indicating that the acid protonation significantly modified the microstructure of the material, resulting in the disappearance of the (100) crystal plane of the sample. The samples P-g-C_3_N_4_, P-g-C_3_N_4_/α-SiC and P-g-C_3_N_4_/β-SiC both showed similar changes.

**Fig. 2:**
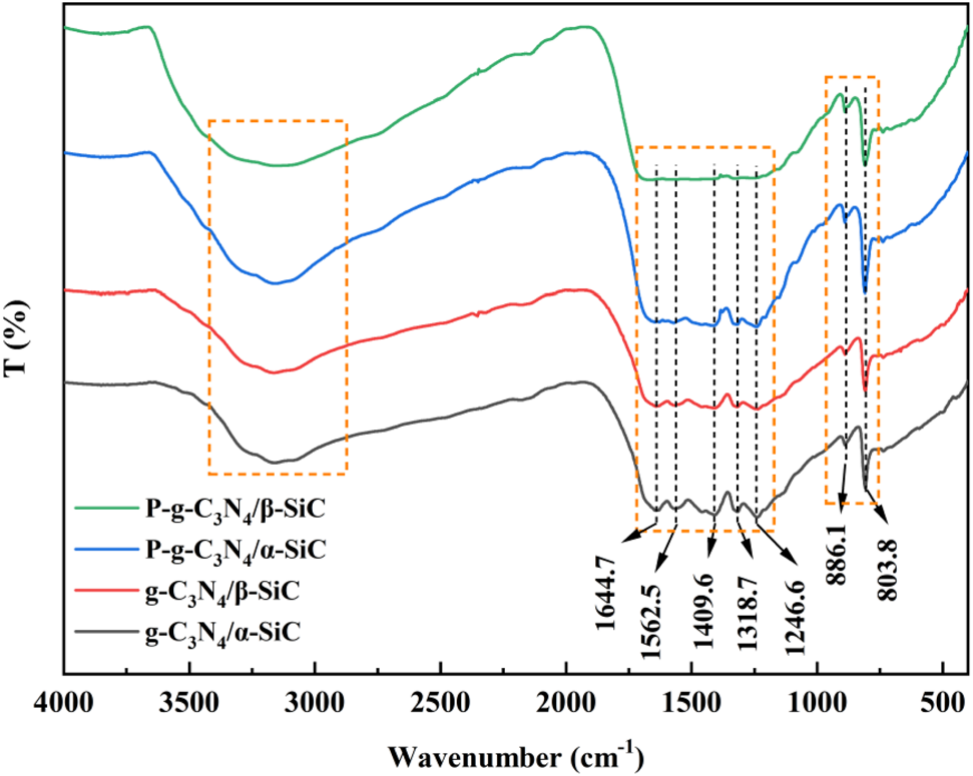
The FT-IR spectra of g-C_3_N_4_/α-SiC, g-C_3_N_4_/β-SiC, P-g-C_3_N_4_/α-SiC and P-g-C_3_N_4_/β-SiC, respectively.

The infrared spectrum of the bulk g-C_3_N_4_ and all composite materials is shown in Fig. 2. All samples maintain the same structure as the classic g-C_3_N_4_. The basic structure of the protonated and unprotonated composite samples did not change significantly in the range of 3000–3500 cm^−1^ (representing the stretching vibration of the N–H functional group) and 1200–1700 cm^−1^ (representing the stretching vibration of C–N–HC and C=N functional group in the triazine ring skeleton). It is worth noting that the absorption peak near 890 cm^−1^ correspond to the cross-linked heptazine deformation mode and the peak of 803.8 cm^−1^ is attributed to the typical tristriazine ring structure.^43^ When the composite material is treated with HCl, the characteristic peak appears to be significantly enhanced, indicating that acid protonation has a significant modification effect on the triazine ring structure of the composite material.^44^ It is comprehensively shown that the doping of silicon carbide does not change the basic characteristic peaks of g-C_3_N_4_, and the protonation of HCl modifies the triazine structure of the material.

**Fig. 3:**
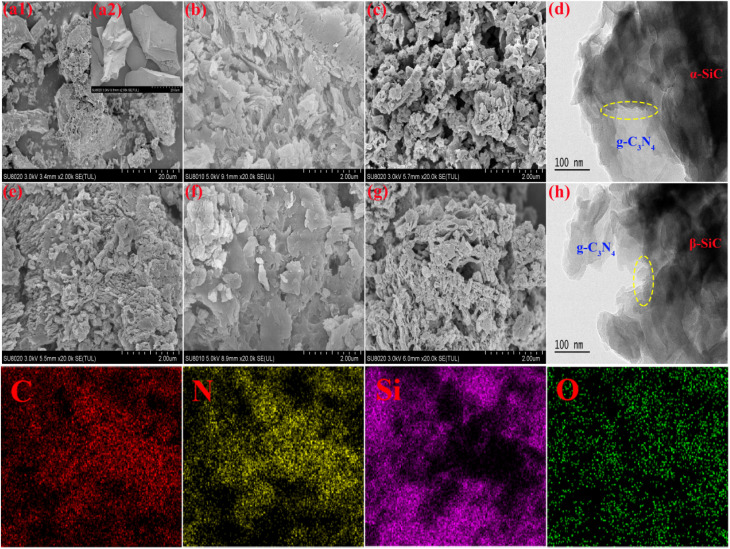
The SEM images of β-SiC (a1), α-SiC (a2), P-g-C_3_N_4_ (e), g-C_3_N_4_/α-SiC (b), P-g-C_3_N_4_/α-SiC (c), g-C_3_N_4_/β-SiC (f), P-g-C_3_N_4_/β-SiC (g) and HRTEM image of P-g-C_3_N_4_/α-SiC (d), P-g-C_3_N_4_/β-SiC (h), and the energy dispersive X-ray spectroscopy (EDS mapping) spectrum respectively.

The morphology and the microstructure of the α-SiC, β-SiC, P-g-C_3_N_4_, g-C_3_N_4_/α-SiC, g-C_3_N_4_/β-SiC, P-g-C_3_N_4_/α-SiC and P-g-C_3_N_4_/β-SiC were investigated by SEM and HRTEM, as shown in Fig. 3. The structures of both β-SiC and α-SiC are granular, with dense and smooth structures without pores (as shown in Fig. 3a1 and a2). The surface structure of protonated g-C_3_N_4_ is relatively dense and accompanied by layered accumulation, and there is a certain pore structure (Fig. 3e). Compared with P-g-C_3_N_4_, a large number of cracks appeared on the surface of g-C_3_N_4_/α-SiC (Fig. 3b) composites and accompanied by an increase in pore structure, some of the structures still showed layered accumulation. Similarly, the g-C_3_N_4_/β-SiC (Fig. 3f) material also has a similar structure. In addition, compared with Fig. 3b, e and f, the overall structure of P-g-C_3_N_4_/α-SiC (Fig. 3c) and P-g-C_3_N_4_/β-SiC (Fig. 3g) has more obvious dispersion, more pores and more wrinkles, and the complete sheet structure is no longer maintained. This shows that the structure of the protonated composite material is looser, which contributes to the increase of the specific surface area and the exposure of active sites. It is worth noting that the even deposition of SiC nanoparticles on surface of g-C_3_N_4_ in SEM images is responsible for the abatement of g-C_3_N_4_ diffraction peaks in XRD patterns. It can be seen from HRTEM that g-C_3_N_4_ exists in amorphous form, with SiC in dark contrast and g-C_3_N_4_ in bright contrast, and the carbon nitride, and α-SiC, β-SiC keep excellent contact with each other (Fig. 3d and h). The materials g-C_3_N_4_ and SiC combine at the interface of the two and are uniformly dispersed and closely connected, which further means that the two semiconductors are in close contact. This close contact not only makes P-g-C_3_N_4_/α-SiC and P-g-C_3_N_4_/β-SiC samples more stable, and can promote the rapid separation and migration of photogenerated carriers. In addition, due to the role of acid protonation, the degree of material defects increases, which is more conducive to enhancing the photocatalytic activity of materials. Besides, the EDS mapping spectrum shows that there are related C, N, Si and O elements in the P-g-C_3_N_4_/β-SiC catalyst.

**Fig. 4:**
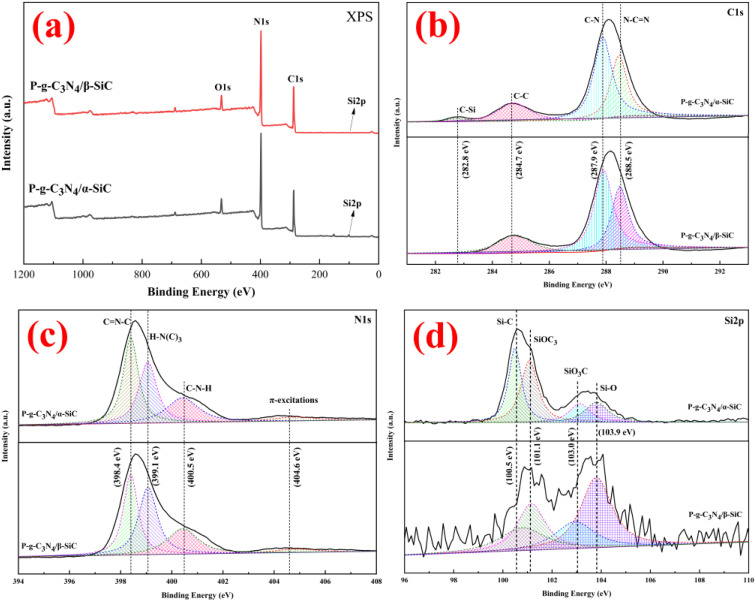
The XPS spectra of P-g-C__3__N__4__/α-SiC and P-g-C__3__N__4__/β-SiC ((a) XPS full spectrum; (b) C 1s; (c) N 1s; (d) Si 2p, respectively).

Fig. 4 shows sample P-g-C_3_N_4_/α-SiC and P-g-C_3_N_4_/β-SiC fit spectra of C 1s, N 1s, and Si 2p of XPS. The survey spectrum shows that sample P-g-C_3_N_4_/α-SiC and P-g-C_3_N_4_/β-SiC are mainly composed of C, N, O, and Si elements (Fig. 4a), of which oxygen is mainly provided by free water on the sample surface. As can be seen from C 1s (Fig. 4b), samples P-g-C_3_N_4_/α-SiC and P-g-C_3_N_4_/β-SiC show four characteristic peaks by fitting the spectrogram, and the binding energy is attributed to the C–C bond of amorphous carbon at 284.7 eV; The peak at 287.9 eV belongs to C–N coordination bond; the strong peak at 288.5 eV is attributed to the N–C=N bond of sp^2^ hybridization in the aromatic ring structure; the weak peak at 282.8 eV belongs to the hybrid C–Si bond. The N 1s spectrogram shows four peak positions through fitting analysis for samples P-g-C_3_N_4_/α-SiC and P-g-C_3_N_4_/β-SiC the binding energies of which are all around 398.4 eV, 399.1 eV, 400.5 eV, and 404.6 eV (Fig. 4c), corresponding to C=N–C, H–N(C)__3__, C–N–H and π excitations, respectively. The C=N–C correspond to sp^2^-hybridized nitrogen in the aromatic rings, which can contribute to the band gap absorption, while the sp^3^ hybrid H–N(C)_3_ and C–N–H atoms represent the sum of protons, which can identify the polymerization of melamine and reflect the structural defects of the composite.^45^ In addition, the ratio of the C=N–C peak area to the sum of H–N(C)_3_ and C–N–H peak areas may be related to the degree of polymerization of acid-protonated materials.^46^ Wherein, sample P-g-C_3_N_4_/β-SiC the polymerization degree is higher than P-g-C_3_N_4_/α-SiC, indicating that the photocatalytic performance of the former may be better than that of the latter, which is due to the increase of polymerization degree is conducive to the improvement of photocatalytic performance. The fitting analysis of the Si 2p spectrum is shown in Fig. 4d. The binding energies of samples P-g-C_3_N_4_/α-SiC and P-g-C_3_N_4_/β-SiC are 100.5 eV, 101.1 eV, 103.0 eV, and 103.9 eV, respectively, corresponding to Si–C, SiOC_3_, SiO_3_C and Si–O.^47^

**Fig. 5:**
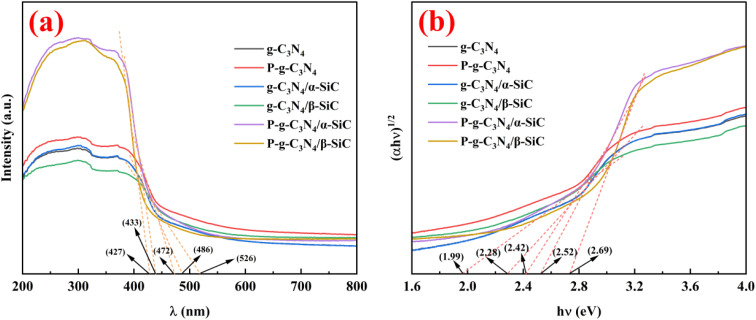
The UV-vis-DRS spectra (a) and band gap energy (b) of composite materials, respectively.

The optical properties of the samples were studied by UV visible diffuse reflectance spectroscopy, as shown in Fig. 5. All samples showed a visible light response (Fig. 5a). Wherein, sample g-C_3_N_4_/α-SiC and g-C_3_N_4_/β-SiC have similar characteristic absorption edges with bulk g-C_3_N_4_, which indicates that g-C_3_N_4_ and SiC retain the basic crystal skeleton structure after recombination. However, the absorption wavelength of sample g-C_3_N_4_/α-SiC in visible light is about 486 nm (the corresponding band gap is 2.28 eV). In comparison g-C_3_N_4_/β-SiC has a wider absorption wavelength (about 526 nm, the corresponding band gap is 1.99 eV) in the visible light region. This shows that the introduction of β-SiC can cause more positive effects on the optical response of composites than that of α-SiC. Compared with g-C_3_N_4_, g-C_3_N_4_/α-SiC and g-C_3_N_4_/β-SiC, the intrinsic absorption edge of sample P-g-C_3_N_4_ shifted from 486 nm to 472 nm, and P-g-C_3_N_4_/α-SiC shifted from 486 nm to 433 nm, and P-g-C_3_N_4_/β-SiC shifted from 526 nm to 427 nm, respectively. This blue shift phenomenon may be attributed to the weakening of the π-conjugated system of crystal materials caused by acid protonation modification.^48^ From the Kubelka–Munk function^49^ (*i.e.*, drawing (*αhν*)^1/2^ relative to photon energy diagram (*hν*), where *α* is the light absorption index, *ν* is the light frequency, *h* is Planck constant) to determine that the energy band gaps of samples g-C_3_N_4_, P-g-C_3_N_4_, g-C_3_N_4_/α-SiC, g-C_3_N_4_/β-SiC, P-g-C_3_N_4_/α-SiC and P-g-C_3_N_4_/β-SiC are 2.28 eV, 2.42 eV, 2.28 eV, 1.99 eV, 2.52 eV, and 2.69 eV, respectively (as shown in Fig. 5b). This further indicates that the blue shift can be attributed to the weakening of π-conjugated system and the corresponding quantum confinement effect. Similarly, compared with g-C_3_N_4_/α-SiC and g-C_3_N_4_/β-SiC, samples P-g-C_3_N_4_/α-SiC and P-g-C_3_N_4_/β-SiC show enhanced visible light absorption in the range of 427 nm–800 nm, which almost covers most areas of the visible spectrum. Generally speaking, enhancing the absorption intensity of visible light can improve the utilization rate of light and may improve the photocatalytic performance of materials.^50^

**Fig. 6:**
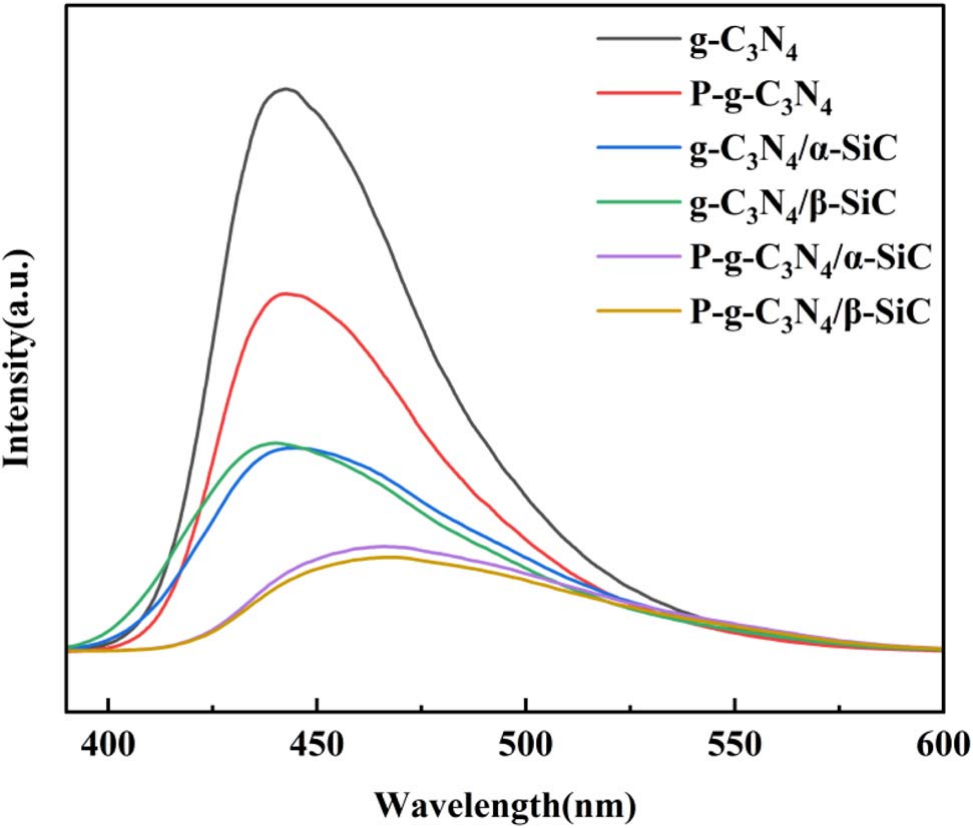
The PL spectra of g-C_3_N_4_, P-g-C_3_N_4_, g-C_3_N_4_/α-SiC, g-C_3_N_4_/β-SiC, P-g-C_3_N_4_/α-SiC and P-g-C_3_N_4_/β-SiC, respectively.

The photogenerated charge transfer process in the photocatalytic reaction process was analyzed by PL, as shown in Fig. 6. The fluorescence spectral analysis is considered to be one of the most powerful technologies to characterize the photogenerated electron recombination process, in which higher PL intensity usually means higher photoinduced charge recombination. For the g-C_3_N_4_, the strong luminescence peak near 450 nm indicates the rapid recombination of the charge carrier induced by light. The PL peak intensity of samples P-g-C_3_N_4_, g-C_3_N_4_/α-SiC, g-C_3_N_4_/β-SiC, P-g-C_3_N_4_/α-SiC and P-g-C_3_N_4_/β-SiC decreased significantly, indicating that the recombination of photogenerated electron hole pairs was effectively suppressed, and the photocatalytic performance would be improved. Similarly, the PL peak of P-g-C_3_N_4_/α-SiC and P-g-C_3_N_4_/β-SiC has a significant red shift, and its wavelength has a red shift to around 462 nm. Among them, the change of the state density of carriers is the main reason for the redshift of the absorption peak. When the concentration of carriers increases, the state density will also increase, resulting in the change of the band structure, which further indicates that the visible light catalytic response of the acid-protonated composites has been significantly improved.^51^ The PL peak intensity of sample P-g-C_3_N_4_/β-SiC is the lowest, which indicates that the recombination of g-C_3_N_4_ and β-SiC and the acid protonation have a positive impact on the effective separation of photogenerated electron–hole pairs and the migration of photogenerated carriers. In addition, this also shows that the composite effect of β-SiC is better than that of α-SiC, and the composite effect of SiC and g-C_3_N_4_ is better than that of acid protonation. Finally, the SiC and g-C_3_N_4_ have good band matching and form heterojunction, which provides a suitable driving force for the separation and transmission of photogenerated electron hole pairs.

**Fig. 7:**
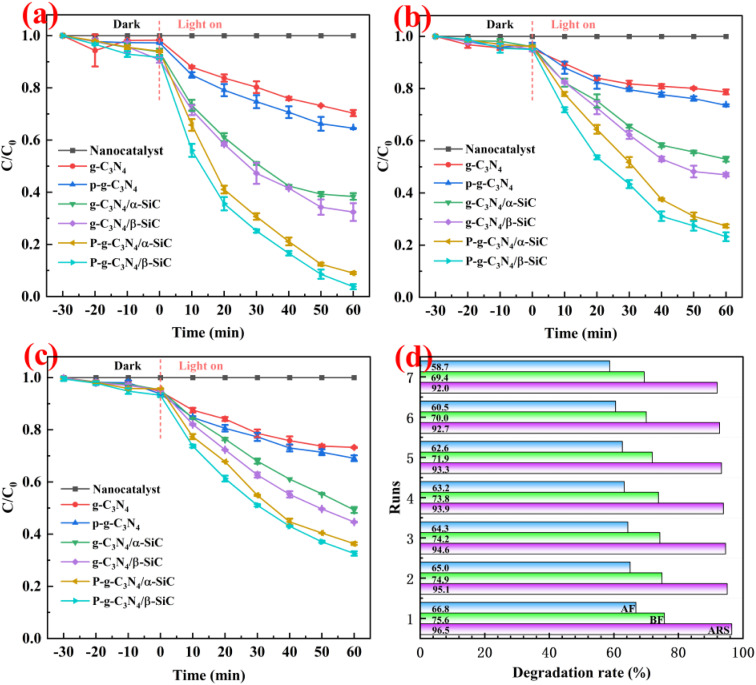
The mean degradation efficiency of catalyst for different dye wastewater ((a): alizarin red; (b): basic fuchsin; (c): acid fuchsin) and catalyst reusability (d).

Through two groups of degradation parallel experiments of alizarin red, basic fuchsin and acid fuchsin dyes, the photocatalytic activity of the material was evaluated (as shown in Fig. 7a–c). It can be seen that sample g-C_3_N_4_ and P-g-C_3_N_4_ has degradation effect on three different dye wastewater, but the degradation rate is low. The mean degradation rate rate of g-C_3_N_4_/α-SiC and g-C_3_N_4_/β-SiC to alizarin red is 61.6% and 67.6% within 60 min, while the mean degradation rate of basic fuchsin and acid fuchsin is 47.1%, 53.0% and 50.7%, 55.4% within 60 min, respectively. It is important that the mean degradation rate of P-g-C_3_N_4_/α-SiC and P-g-C_3_N_4_/β-SiC to alizarin red is 91.0% and 96.2% within 60 min, while the mean degradation rate of basic fuchsin and acid fuchsin is 72.7%, 76.8% and 63.7%, 67.4% within 60 min, respectively. It is worth noting that the photocatalytic performance of P-g-C_3_N_4_/α-SiC and P-g-C_3_N_4_/β-SiC in the catalytic degradation of three dyes is better than that of g-C_3_N_4_/α-SiC and g-C_3_N_4_/β-SiC, of which sample P-g-C_3_N_4_/β-SiC has the best performance, followed by P-g-C_3_N_4_/α-SiC. In addition, in terms of time and degradation rate evaluation, the degradation rate of alizarin red by samples P-g-C_3_N_4_/α-SiC and P-g-C_3_N_4_/β-SiC is better than that of basic fuchsin and acid fuchsin, and the degradation rate of acid fuchsin wastewater is the lowest. It may be attributed to the intrinsic structural characteristics of fuchsin acid and the hydrogen bond effect between different groups. In addition, the important indicators for evaluating excellent catalyst include good catalytic performance and excellent cycle life cycle. In view of this, we studied the catalytic cycle of the best catalyst P-g-C_3_N_4_/β-SiC for three different dyes through the first set of degradation experiments (as shown in Fig. 7d). The results showed that the activity of the catalyst was almost stable after 7 times of recycling. After seven cycles, the degradation rates were 92.0% of alizarin red, 69.4% of basic fuchsin and 58.7% of acid fuchsin.

**Fig. 8:**
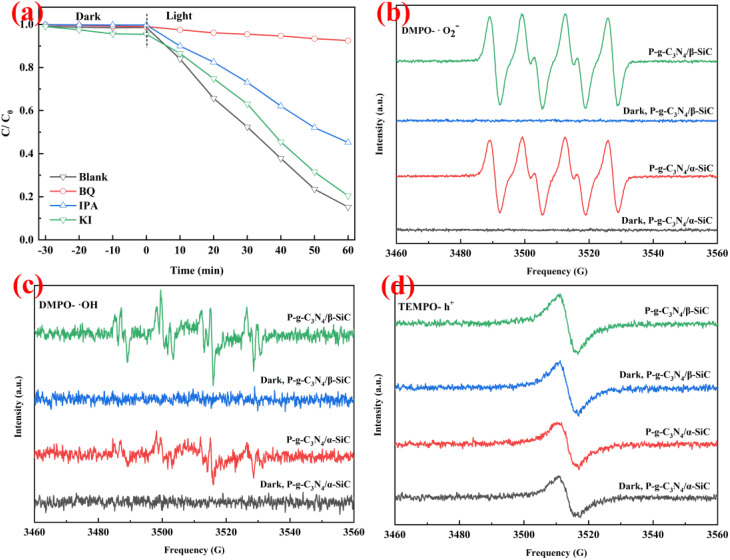
(a): The plots of photogenerated carriers trapping in the system of photodegradation of ARS by P-g-C_3_N_4_/β-SiC under visible light. The ESR spectra of radical adducts trapped by DMPO (˙O_2_^−^ and ˙OH) and TEMPO (h^+^) in the dark and under visible light irradiation: (b) in methanol dispersion of P-g-C_3_N_4_/α-SiC and P-g-C_3_N_4_/β-SiC for DMPO-˙O_2_^−^. (c) In aqueous dispersion of P-g-C_3_N_4_/α-SiC and P-g-C_3_N_4_/β-SiC for DMPO-˙OH and (d) in aqueous dispersion of P-g-C_3_N_4_/α-SiC and P-g-C_3_N_4_/β-SiC for TEMPO-h^+^.

To explore the primary active species generated in the reaction system, trapping experiment of active species was conducted as shown in Fig. 8a. The *p*-benzoquinone, isopropanol, and potassium iodide as capture agents for superoxide radicals (˙O_2_^−^), hydroxyl radicals (˙OH), and holes (h^+^), respectively. When *p*-benzoquinone as the scavenger for ˙O_2_^−^ was added, the photo-degradation efficiency of ARS was significantly reduced, indicating that the ˙O_2_^−^ played a major catalytic role in the photocatalytic reaction. When potassium iodide was added as a scavenger for h^+^, the photodegradation efficiency of ARS decreased slightly, but the decline was small, indicating that h^+^ had a certain auxiliary catalytic effect in the photocatalytic reaction. It is worth noting that when isopropanol is added as a scavenger of ˙OH, the photodegradation efficiency of ARS also decreases to a certain extent. This shows that ˙OH also has an excellent catalytic effect in photocatalytic reactions. This result shows that ˙O_2_^−^ and ˙OH produced in the photocatalytic system play a leading role in the photocatalytic degradation reaction, while h^+^ only plays an auxiliary role.

In order to further confirm the main active species of P-g-C_3_N_4_/α-SiC and P-g-C_3_N_4_/β-SiC during the photocatalytic reaction, the ESR spin-trap with DMPO and TEMPO techniques were performed under visible light irradiation, as shown in Fig. 8b–d. We observe that the samples P-g-C_3_N_4_/α-SiC and P-g-C_3_N_4_/β-SiC have obvious DMPO-˙O_2_^−^ characteristic peaks under visible light in Fig. 8b. This result indicates that ˙O_2_^−^ plays an important role in the photocatalytic reaction, which further confirms the experimental results in Fig. 8a. It is worth noting that the DMPO-˙O_2_^−^ characteristic peak intensity of the sample P-g-C_3_N_4_/β-SiC is significantly higher than that of the sample P-g-C_3_N_4_/α-SiC. However, all samples produced no signal in the dark. The characteristic peak of DMPO-˙OH can be seen in Fig. 8c. The ˙OH signal generated by the sample P-g-C_3_N_4_/α-SiC is relatively weak, while the ˙OH signal corresponding to the sample P-g-C_3_N_4_/β-SiC is stronger. This shows that ˙OH has played a certain catalytic role in the photocatalytic degradation reaction, and the catalytic performance of the sample P-g-C_3_N_4_/β-SiC is better than that of P-g-C_3_N_4_/α-SiC. Similarly, none of the samples produced a signal under the dark reaction condition.

**Fig. 9:**
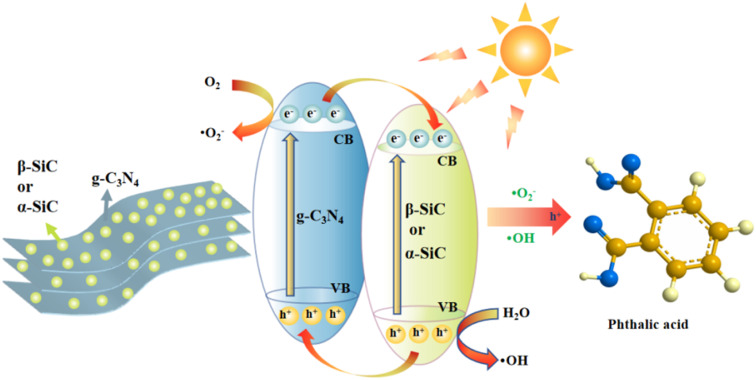
Schematic illustration of the mechanism for the photocatalytic degradation of ARS by P-g-C_3_N_4_/β-SiC or P-g-C_3_N_4_/α-SiC under visible light.

According to the above discussions, we propose a photocatalytic mechanism to better explain the detailed process of alizarin red wastewater under the photocatalytic degradation (as shown in Fig. 9). After acid protonation, SiC particles can closely cover the surface of layered g-C_3_N_4_ and form strong interaction. In addition, g-C_3_N_4_ and SiC have good band matching and can form heterojunction. In the system of catalytic degradation of alizarin red, the strong oxidizing ˙O_2_^−^ and ˙OH groups formed contact and react with alizarin red through charge transfer and transmission, and finally form colorless, water-soluble phthalic acid and other small molecule organics.

## 3. Conclusion

In summary, the composite materials (P-g-C_3_N_4_/β-SiC and P-g-C_3_N_4_/α-SiC) prepared by acid protonation showed excellent catalytic activity in the evaluation of photocatalytic degradation of ARS, BF and AF dye wastewater. Although the catalytic degradation performance of sample P-g-C_3_N_4_/β-SiC for three dyes is higher than that of P-g-C_3_N_4_/α-SiC, the degradation performance of these two materials is significantly better than that of g-C_3_N_4_/α-SiC and g-C_3_N_4_/β-SiC. In addition, the characterization of UV-vis-DRS and PL showed that material P-g-C_3_N_4_/β-SiC had the best performance, followed by P-g-C_3_N_4_/α-SiC. However, it cannot be ignored that the two catalysts prepared in this work have excellent photocatalytic performance.

## 4. Experimental section

All chemicals were of analytical grade and obtained from Beijing Innochem Technology Co., Ltd., and used without further purification.

### 4.1 Preparation of SiC material

The sodium silicate and ferric nitrate are completely dissolved in 30 mL of deionized water (*n*(Fe) : *n*(Si) = 0.01), and Taixi coal (particle size is 120 mesh, *m*(Taixi coal) : *m*(silicon sodium) = 5 : 1, produced in Ningxia, China). The mixed solution was magnetically stirred at 90 °C until everything turned into a gel. The gel-like product is transferred to an infrared drying oven at 100 °C for 24 hours, and the sample is crushed to obtain a silicon carbide precursor. Next, the precursor was put into an alumina tube high-temperature furnace and calcined at 1450 °C for 6 hours in an argon atmosphere to prepare a crude silicon carbide product. After the reaction, the product was calcined in an air atmosphere at 850 °C for 5 hours to remove residual carbon. Then use mixed acid (V(HF) : V(HCl) = 2 : 3) to soak and magnetically stir for 12 h to remove residual trace metal and inorganic salts in the product. Then, it was washed with water to neutrality, centrifuged and dried to obtain a pure β-SiC sample. In addition, the pure crystalline phase α-SiC material is purchased commercially (Henan Ming Maita New Material Technology Co, Ltd. China).

### 4.2 Preparation of carbon nitride composite silicon carbide material

The pure bulk g-C_3_N_4_ was synthesized *via* calcination of melamine (5 g) and urea (5 g) in a corundum crucible with a cover at 550 °C for 2 h. After cooling to room temperature, a yellow g-C_3_N_4_ product was obtained. The melamine and urea (mixed mass ratio 1 : 1) and silicon carbide (β-SiC or α-SiC; mass ratio 10 : 1) were placed in a round bottom flask containing 30 mL methanol. Stir continuously at room temperature for 30 minutes and then heat to 100 °C to distill and recover methanol to obtain a mixed powder sample. The sample was put into an alumina crucible and heated at 2 °C min^−1^ to 350 °C and kept for 1 h; then heated at 5 °C min^−1^ to 550 °C and kept for 2 h, the composite material g-C_3_N_4_/α-SiC and g-C_3_N_4_/β-SiC were obtained.

### 4.3 Preparation of protonated composite sample

**Fig. 10:**
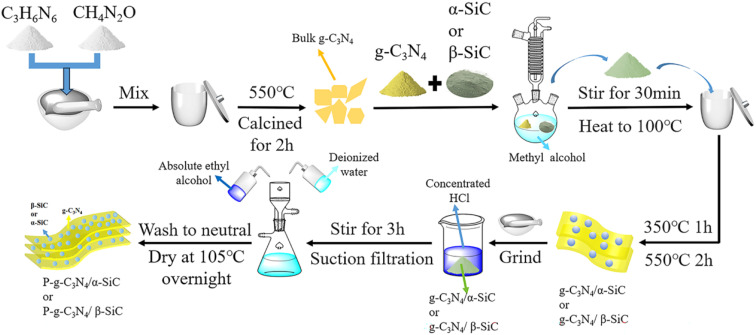
The schematic diagram of the preparation process of photocatalyst samples.

Using the literature method,^49^ the add 3 g of g-C_3_N_4_/α-SiC or g-C_3_N_4_/β-SiC to 30 mL concentrated hydrochloric acid (mass fraction 37%). Stir vigorously at room temperature for 3 h, then suction filter, continue to wash with deionized water and absolute ethanol until neutral, and dry overnight at 105 °C to obtain protonated g-C_3_N_4_/α-SiC or g-C_3_N_4_/β-SiC composites, and were marked as P-g-C_3_N_4_/α-SiC and P-g-C_3_N_4_/β-SiC, respectively. The simple preparation process is shown in Fig. 10.

### 4.4 Characterization

The X-ray diffraction (XRD) was performed on a Bruker-AXS D8 Advance using Cu Kα radiation (40 kV/40 mA). The Fourier transform infrared (FTIR) spectra were recorded on a Bruker spectrometer (AVANCE III 400) over the frequency range 400–4000 cm^−1^ at a resolution of 2 cm^−1^. The scanning electron microscopy (SEM) images were collected on a FEI verous 460 instrument. The FEI Tecnai G2 F20 electron microscope (FEI, USA) was used for transmission electron microscopy (TEM) and high-resolution TEM (HRTEM) analyses. The UV-vis diffuse reflectance spectra (UV-vis-DRS) were collected on a UV2000 UV-vis spectrophotometer. The spectra were recorded at room temperature in air from 200 to 800 nm. The X-ray photoelectron spectroscopy (XPS) data was acquired on a ESCALAB 250Xi (ThermoFisher, USA) equipped with a Al Kα X-ray source (*hν* = 1486.6 eV) operating at 150 W. The photoluminescence (PL) spectra were measured at room temperature on an F-7100 fluorescence spectrophotometer (Hitachi, Japan) with an excitation wavelength of 385 nm. The electron spin resonance spectra were recorded on an ESR spectrometer (Bruker A300, Germany). A TU-1901 dual-beam UV-vis spectrophotometer (UV-vis, Purse General Instrument Co., Beijing, China) was used to collect the photocatalytic reaction data at room temperature. The use Bilon-GHX-II photochemical reaction instrument for photocatalytic reaction test (Bilon Instrument Company, Shanghai, China).

### 4.5 Evaluation of photocatalytic degradation of dye wastewater

In order to investigate the photocatalytic activity of samples g-C_3_N_4_/α-SiC, g-C_3_N_4_/β-SiC, P-g-C_3_N_4_/α-SiC and P-g-C_3_N_4_/β-SiC, alizarin red, basic fuchsin and acid fuchsin dyes were used as the photodegradation test objects. The visible light source is 300 W metal halide lamp. First, disperse 40 mg catalyst in 80 mL, 40 mg L^−1^ dye aqueous solution for dark reaction for 30 min, so that the catalyst in the solution can reach adsorption equilibrium with the solution. Then turn on the light source to react and take samples every 15 minutes. After the solvent sample is centrifuged, the supernatant is taken, and the absorbance at 420 nm, 543 nm and 545 nm is measured with an ultraviolet visible spectrophotometer.

### 4.6 Trapping experiment of catalytic active species

The 0.5 mmol of *p*-benzoquinone (BQ), 0.5 mmol of isopropanol (IPA) and 0.5 mmol of disodium edetate dihydrate (EDTA-2Na) were added to ARS, AF and BF dyes as scavengers for photocatalytic degradation experiments. No scavenger was added as a blank control experiment.

## Conflicts of interest

The authors declare that they have no known competing financial interests or personal relationships that could have appeared to influence the work reported in this work.

## Supplementary Material
